# AI literacy is directly associated with positive attitudes and indirectly associated with negative attitudes through AI anxiety: a cross-sectional study of preservice teachers

**DOI:** 10.3389/fpsyg.2026.1946898

**Published:** 2026-10-22

**Authors:** İsmail Satmaz, Mehmet Adıyaman

**Affiliations:** 1 Department of Educational Sciences, Faculty of Education, Çanakkale Onsekiz Mart University, Çanakkale, Türkiye; 2 School of Graduate Studies, Çanakkale Onsekiz Mart University, Çanakkale, Türkiye

**Keywords:** AI anxiety, AI literacy, attitudes toward AI, preservice teachers, teacher education

## Abstract

**Introduction:**

Positive and negative attitudes toward artificial intelligence (AI) may have partly distinct psychological correlates. This cross-sectional study examined whether AI anxiety statistically accounted for associations between AI literacy and positive and negative attitudes toward AI among preservice teachers.

**Methods:**

Participants were 335 preservice teachers enrolled in a faculty of education in Türkiye. Data were collected using the Artificial Intelligence Literacy Scale, Artificial Intelligence Anxiety Scale, Artificial Intelligence Attitude Scale, and a personal information form. Two indirect-effect models were estimated using PROCESS Model 4 with 5,000 bootstrap resamples, controlling for prior AI training.

**Results:**

AI literacy was negatively associated with AI anxiety and positively associated with positive attitudes toward AI. AI anxiety was positively associated with negative attitudes but was not significantly associated with positive attitudes. The indirect association through AI anxiety was significant for negative attitudes but not for positive attitudes, and a direct bootstrap contrast confirmed that the two indirect effects differed significantly (B = −0.118, 95% CI [−0.201, −0.039]). Prior AI training was not significantly associated with either attitude dimension. A sensitivity analysis excluding the three reverse-worded AI literacy items reproduced this pattern.

**Discussion:**

The findings indicate that positive and negative attitudes toward AI may have partly distinct psychological correlates: AI literacy is directly related to positive evaluations, whereas its association with negative evaluations is statistically linked to AI anxiety. Because the data are cross-sectional, the indirect effects should not be interpreted as evidence of causal mediation.

## 1 Introduction

The growing use of artificial intelligence (AI) in education extends beyond technological and pedagogical change; it also creates psychological adaptation challenges involving knowledge, uncertainty, anxiety, perceived risk, and evaluative attitudes. AI-supported systems can provide personalized guidance, feedback, assessment, and decision support (Hwang et al., 2020), yet these systems may also evoke concerns about control, employment, privacy, fairness, and human agency. For preservice teachers, such cognitive and affective responses are consequential because they may shape how future educators interpret and approach AI-supported learning environments. Zawacki-Richter et al. (2019) further noted that educators and the pedagogical and ethical implications of AI have received limited attention in parts of the higher-education literature.

For preservice teachers, AI integration should not be viewed merely as a technical skill. AI is reshaping teacher-student-technology relationships and prompting reconsideration of teachers’ roles. Guan et al. (2025) found that preservice teachers often viewed AI as a mechanically used tool rather than a dynamic educational partner and needed a deeper understanding of AI fundamentals and ethical issues. Lucas et al. (2025) similarly showed that preservice teachers were not fully prepared for AI integration and argued that teacher education programs should be reviewed in terms of AI readiness. These studies indicate that readiness for AI extends beyond frequency of use and should be considered alongside knowledge, perceptions, competence, trust, and educational meaning-making.

Within this framework, AI literacy is a relevant individual-difference variable for understanding how preservice teachers perceive and evaluate AI. Long and Magerko (2020) define AI literacy as a set of competencies that enable individuals to interact effectively with AI and critically evaluate AI systems. Ng et al. (2021) conceptualized AI literacy in terms of knowing and understanding, using and applying, evaluating and creating, and recognizing ethical issues. This perspective shows that AI literacy cannot be reduced to technical knowledge alone; rather, it represents a multidimensional competence related to pedagogical decision-making, critical evaluation, and ethical awareness.

Recent research also identifies AI literacy as an important competence in teacher education. Casal-Otero et al. (2023) argued that AI literacy should be addressed through an interdisciplinary, competence-based approach that actively involves teachers. Pei et al. (2025) identified notable gaps in preservice teachers’ understanding of AI mechanisms and found that AI literacy was associated with engagement, perceptions, and ethical evaluations. AI literacy may therefore provide an important foundation for more informed, realistic, and pedagogically grounded evaluations of AI among preservice teachers.

AI literacy may also be associated with attitudes toward AI. As preservice teachers become better able to understand, use, and evaluate AI systems, they may develop a clearer understanding of the educational affordances and limitations of these systems. Greater understanding of instructional support, personalized learning, feedback, assessment, and learning analytics may be associated with more positive attitudes toward AI. A clearer understanding of AI mechanisms and ethical implications may also reduce the tendency to perceive AI as unknown or uncontrollable. Accordingly, greater AI literacy may be associated with more positive attitudes and lower AI anxiety.

AI anxiety may be an important psychological variable in this relationship. Wang and Wang (2022) define AI anxiety as a general fear or anxiety response that may hinder individuals’ interaction with AI. They conceptualized AI anxiety across several dimensions, including learning, job replacement, sociotechnical blindness, and AI configuration. Limited knowledge about AI, difficulty understanding how AI systems operate, and uncertainty about their consequences for work and society may contribute to anxiety. Conversely, greater access to AI-related knowledge and learning opportunities may be associated with lower anxiety and more favorable acceptance-related evaluations.

Research with preservice teachers likewise indicates that AI anxiety is related to attitudes toward AI. Hopcan et al. (2024) found that preservice teachers did not report marked anxiety about learning AI but expressed concerns about its effects on employment and social life. The same study reported that some dimensions of AI anxiety decreased as personal experience increased, suggesting that experience, familiarity, and knowledge may be associated with lower anxiety. Hopcan et al. (2024) also identified significant relationships between AI anxiety and attitudes toward machine learning, further indicating that anxiety is related to how preservice teachers evaluate AI technologies.

AI anxiety may be particularly relevant to negative attitudes. Higher anxiety may be associated with greater perceived risks related to job loss, privacy, security, algorithmic bias, social inequality, and human autonomy. Hopcan et al. (2024) reported that individuals with higher AI anxiety may develop more negative evaluations of machine learning. Wang and Wang (2022) conceptualized AI anxiety as a belief-related variable associated with behavioral intentions and subsequent behavior. From this perspective, AI anxiety is more than an affective response; it may also be related to how individuals evaluate and approach AI.

An important psychological feature of attitudes toward AI is that positive and negative evaluations are not necessarily opposite endpoints of a single continuum. Individuals may recognize the personal or social utility of AI while simultaneously experiencing fear or reservations about its ethical, professional, and social consequences. Schepman and Rodway (2020) identified distinct positive and negative dimensions of general attitudes toward AI: the positive dimension reflected perceived utility, whereas the negative dimension reflected anxiety and concern. Sindermann et al. (2021) similarly distinguished acceptance-related and fear-related evaluations. This dual-evaluation perspective allows positive appraisals and negative affective responses to coexist and provides a psychological basis for expecting the two attitude dimensions to have different correlates.

This distinction raises the question of whether AI literacy is related to positive and negative attitudes through the same psychological pathway. Greater literacy may be directly associated with more positive evaluations because greater knowledge and understanding provide a broader basis for appraising AI and its potential uses. Its association with negative evaluations may instead be statistically linked to lower anxiety arising from reduced uncertainty about how AI systems operate and what consequences they may have. Accordingly, this study tests whether AI anxiety accounts for an indirect association between AI literacy and each attitude dimension. The term indirect association is used deliberately because all variables were measured at one time point; therefore, the analysis cannot establish temporal order or causal mediation. Specifically, the study examines whether (a) AI literacy is negatively associated with AI anxiety, (b) AI anxiety is differentially associated with positive and negative attitudes, and (c) the corresponding bootstrap indirect effects differ across the two attitude dimensions.

The following hypotheses were tested in this study:

*H_1_*: AI literacy is negatively associated with AI anxiety.

*H_2_*: AI anxiety is positively associated with negative attitudes toward AI.

*H_3_*: AI anxiety is negatively associated with positive attitudes toward AI.

*H_4a_*: AI anxiety statistically accounts for a significant indirect association between AI literacy and negative attitudes toward AI.

*H_4b_*: AI anxiety statistically accounts for a significant indirect association between AI literacy and positive attitudes toward AI.

*H_5_*: AI literacy is positively associated with positive attitudes toward AI.

## 2 Materials and methods

### 2.1 Research design

This study used a quantitative, cross-sectional correlational design to examine associations among preservice teachers’ AI literacy, AI anxiety, and positive and negative attitudes toward AI. Indirect-effect analysis was used to quantify whether the statistical association between AI literacy and each attitude dimension was accounted for by AI anxiety (Hayes, 2022). Because the design does not establish temporal precedence among the variables, path labels such as predictor, outcome, and indirect effect describe the specified statistical model and do not imply causal effects. Positive and negative attitudes were analyzed as separate outcomes in accordance with the two-dimensional structure of the attitude scale.

### 2.2 Participants

The non-probability volunteer sample consisted of 335 preservice teachers enrolled in one faculty of education in Türkiye. Of these participants, 96 (28.7%) reported prior AI training and 239 (71.3%) reported no prior AI training. Prior AI training was included as a binary covariate in both indirect-effect models. No *a priori* power analysis was conducted before data collection. To characterize the sensitivity of the achieved sample, an achieved-sample sensitivity analysis was performed for multiple linear regression. With *N* = 335, *α* = 0.05, and 80% power, the sample was sufficient to detect an overall effect of approximately *f*^2^ = 0.033 in a model with three predictors. This analysis is reported as a sensitivity assessment of the obtained sample rather than as an *a priori* justification of sample size. The use of a single institution and voluntary participation limits population-level generalization. Demographic characteristics are presented in Table 1. All participants were 18 years of age or older. Age was recorded categorically as 18, 19, 20, 21, 22, or 23 years or older. The age distribution comprised 21 participants aged 18 years, 67 aged 19 years, 104 aged 20 years, 71 aged 21 years, 37 aged 22 years, and 35 aged 23 years or older. No participant was younger than 18 years.

**Table 1:** Demographic information of the participants.

Variable	Group	*n*	%
Gender	Female	208	62.1
	Male	127	37.9
Grade level	First year	70	20.9
	Second year	149	44.5
	Third year	83	24.8
	Fourth year	33	9.9
Department	Social Studies Education	49	14.6
	Science Education	9	2.7
	Elementary Mathematics Education	46	13.7
	English Language Teaching	87	26.0
	Chemistry Education	7	2.1
	Turkish Language Education	71	21.2
	Special Education	66	19.7
Prior AI training experience	Yes	96	28.7
	No	239	71.3

*N* = 335. Percentages may not total 100 due to rounding.

Table 1 summarizes the demographic characteristics of the 335 preservice teachers. The sample included 208 female and 127 male participants. By grade level, 70 participants were in their first year, 149 in their second year, 83 in their third year, and 33 in their fourth year. The largest departmental groups were English Language Teaching, Turkish Language Education, and Special Education. In addition, 96 participants reported prior AI training, whereas 239 reported no prior AI training.

### 2.3 Data collection instruments

Data were collected using a personal information form, the Artificial Intelligence Literacy Scale, the Artificial Intelligence Anxiety Scale, and the Artificial Intelligence Attitude Scale.

#### 2.3.1 Personal information form

The personal information form collected participants’ age, gender, grade level, department, and prior AI training experience. Age was recorded categorically as 18, 19, 20, 21, 22, or 23 years or older.

#### 2.3.2 Artificial intelligence literacy scale

The Artificial Intelligence Literacy Scale was used to assess participants’ AI literacy. The Turkish adaptation by Çelebi et al. (2023) is a 12-item instrument rated on a seven-point Likert scale and comprises four dimensions: awareness, usage, evaluation, and ethics. Three items are reverse-worded. The adaptation study retained the four-factor structure and reported subscale Cronbach’s *α* coefficients ranging from 0.72 to 0.76 and a total-score coefficient of 0.85; it also reported an overall scale score. In the present study, the mean of all 12 items, after reverse scoring the relevant items, was used as a pragmatic observed composite summarizing broader AI literacy. This use should not be interpreted as evidence that the instrument represents a unidimensional latent construct. In the present sample, the overall composite showed acceptable internal consistency (Cronbach’s *α* = 0.744; McDonald’s *ω* = 0.795). Item-level reliability diagnostics showed comparatively weak item-rest correlations for two of the three reverse-worded items (items 2 and 11), whereas the third reverse-worded item had an adequate item-rest correlation. Thus, the lower total-score reliability cannot be attributed uniformly to reverse wording, although wording-related effects may have contributed to the reduced internal consistency. Because reverse-worded items can introduce wording-related method variance, an additional sensitivity analysis was conducted after excluding the three reverse-worded items. This analysis was treated as a robustness check and did not replace the validated 12-item scoring procedure.

#### 2.3.3 Artificial intelligence anxiety scale

The Artificial Intelligence Anxiety Scale was used to assess anxiety toward AI. The Turkish adaptation by Akkaya et al. (2021) consists of 16 items rated on a five-point scale and includes four dimensions: learning, job replacement, sociotechnical blindness, and AI configuration. The adaptation study confirmed the four-factor structure and reported a total Cronbach’s *α* of 0.937, with subscale coefficients ranging from 0.875 to 0.950. Because the hypotheses concerned general AI-related anxiety rather than dimension-specific effects, the overall mean score was used, consistent with the reported total-score reliability. In the present sample, the overall composite showed high internal consistency (Cronbach’s *α* = 0.919; McDonald’s *ω* = 0.919).

#### 2.3.4 Artificial intelligence attitude scale

The Artificial Intelligence Attitude Scale, adapted by Çakan and Akın (2024), was used to assess preservice teachers’ attitudes toward AI. The Turkish form contains 12 items rated on a five-point Likert scale and comprises two subdimensions: positive attitudes toward AI and negative attitudes toward AI. The adaptation study supported this two-factor structure through exploratory and confirmatory factor analyses. The overall Cronbach’s *α* coefficient was 0.826, with coefficients of 0.782 for the positive-attitude subdimension and 0.854 for the negative-attitude subdimension. In the present study, the two subdimensions were analyzed as separate variables. Internal consistency was acceptable for the positive-attitude subscale (Cronbach’s *α* = 0.753; McDonald’s *ω* = 0.757) and good for the negative-attitude subscale (Cronbach’s *α* = 0.849; McDonald’s *ω* = 0.852).

Importantly, the instruments assessed general AI literacy, AI anxiety, and attitudes toward AI rather than attitudes or intentions specifically related to the pedagogical use of AI. Accordingly, the study did not directly measure AI-integration intentions, classroom use, or evaluations of AI-supported teaching practices.

### 2.4 Data collection procedure

Data were collected from preservice teachers enrolled in the participating faculty of education between 29 and 31 May 2026, after ethics approval had been obtained. Eligibility required participants to be at least 18 years old and enrolled as preservice teachers in the participating faculty. Before responding, participants were informed about the study purpose, the voluntary nature of participation, confidentiality, and their right to withdraw; all participants provided informed consent. Participants were recruited through in-person classroom visits, and the paper-and-pencil questionnaires were administered approximately 10 min before or after scheduled classes. No incentives were provided. The number of students approached was not recorded; therefore, a response rate could not be calculated. A total of 335 completed questionnaires were obtained, and none were excluded. One questionnaire was collected from each participant. Completion time was not recorded, and no formal rapid-response or straight-lining exclusion criteria were applied.

### 2.5 Data analysis

Data were analyzed using IBM SPSS Statistics version 27 and the PROCESS macro version 5.0 (Hayes, 2022). Descriptive statistics, skewness, kurtosis, Cronbach’s alpha coefficients, and Pearson correlations were calculated first. Given the relatively strong correlation between AI anxiety and negative attitudes, discriminant validity between these constructs was additionally examined using the heterotrait-monotrait ratio of correlations (HTMT; Henseler et al., 2015). Following the conservative HTMT.85 criterion, values below 0.85 were considered indicative of adequate discriminant validity. The 95% confidence interval for HTMT was estimated using 5,000 percentile bootstrap resamples with a fixed random-number seed of 20260910.

Across the four focal variables, skewness ranged from −0.831 to −0.138 and kurtosis ranged from −0.084 to 2.211. The focal variables were treated as approximately continuous because the analyses used multi-item mean composite scores rather than individual Likert-type items. Each composite represented responses aggregated across multiple items, and the observed score distributions showed sufficient variation without extreme distributional distortion. Regression-based analyses were therefore conducted on the composite scores, with residual, HC3, and outlier sensitivity analyses used to evaluate the robustness of the findings.

Two PROCESS Model 4 analyses were estimated. In both models, AI literacy was specified as the predictor, AI anxiety as the intervening variable, and prior AI training as a covariate. Negative attitude was the outcome in the first model and positive attitude in the second. Indirect effects were evaluated using 5,000 bootstrap resamples and 95% percentile bootstrap confidence intervals; an indirect effect was considered statistically significant when its confidence interval excluded zero. A fixed random-number seed of 20260910 was used for all bootstrap analyses to facilitate reproducibility. Prior AI training was coded as 0 = no prior AI training and 1 = prior AI training and was included as a covariate in both the mediator and outcome equations. Unstandardized coefficients (*B*) are reported as the primary regression estimates, with standardized coefficients (*β*) additionally provided to facilitate effect-size interpretation. Prior AI training was selected as the covariate because it represents previous AI-specific exposure that could plausibly be associated with AI literacy, anxiety, and attitudes. Grade level and department were not included as routine control variables because no specific confounding role was hypothesized for these characteristics; demographic variables were not entered solely because they were available.

Because the study compared the indirect associations for positive and negative attitudes, a direct contrast between the two indirect effects was also estimated. Both attitude outcomes were measured in the same participants and scored on the same 1–5 metric. A difference score was therefore computed by subtracting positive attitude from negative attitude and was analyzed as the outcome in PROCESS Model 4 using the same predictor, mediator, covariate, and 5,000 bootstrap resamples. The resulting indirect effect represents the contrast between the negative- and positive-attitude indirect effects. Given the cross-sectional design, a significant indirect effect was interpreted as a statistical decomposition of associations rather than evidence of a temporally ordered causal mechanism.

Regression assumptions and influential observations were examined using residual plots, standardized and studentized deleted residuals, tolerance and variance inflation factor (VIF) statistics, leverage values, and Cook’s distance. No problematic multicollinearity was detected, and no observation had a Cook’s distance approaching 1. Because the residual diagnostics suggested some departure from constant error variance and several studentized deleted residuals exceeded an absolute value of 3 in the attitude outcome models, two additional robustness checks were conducted. First, the primary models were re-estimated using HC3 heteroskedasticity-consistent standard errors. Second, the models were re-estimated after temporarily excluding observations with absolute studentized deleted residuals greater than 3. These analyses were treated as sensitivity checks; the full *N* = 335 sample remained the basis of the primary analyses. Tables 2, 3 report the primary models estimated using conventional OLS standard errors together with percentile-bootstrap confidence intervals for the indirect effects. HC3 standard errors were used only in the separate robustness analyses reported below and were not substituted for the primary regression standard errors in Tables 2, 3.

**Table 2:** Indirect association between AI literacy and negative attitudes toward AI through AI anxiety.

Model/path	*B*	SE/BootSE	*β*	*t*	*p*	95% CI/Bootstrap 95% CI
Mediator model: AI anxiety						
AI literacy → AI anxiety	−0.1775	0.0569	−0.169	−3.1205	0.0020	[−0.2894, −0.0656]
Prior AI training → AI anxiety	−0.1265	0.0912	−0.075	−1.3867	0.1665	[−0.3059, 0.0529]
Outcome model: Negative attitude						
AI literacy → Negative attitude	−0.0328	0.0463	−0.030	−0.7087	0.4790	[−0.1240, 0.0583]
AI anxiety → Negative attitude	0.6968	0.0441	0.659	15.8088	<0.001	[0.6101, 0.7835]
Prior AI training → Negative attitude	−0.0539	0.0735	−0.030	−0.7335	0.4638	[−0.1984, 0.0906]
Total-effect model: Negative attitude						
AI literacy → Negative attitude	−0.1565	0.0604	−0.141	−2.5903	0.0100	[−0.2754, −0.0377]
Prior AI training → Negative attitude	−0.1420	0.0969	−0.080	−1.4656	0.1437	[−0.3326, 0.0486]
Indirect effect						
AI literacy → AI anxiety → Negative attitude	−0.1237	0.0426	−0.111^*^	–	–	[−0.2065, −0.0398]

*N* = 335. Mediator model: *R*^2^ = 0.0372, *F*(2, 332) = 6.4179, *p* = 0.0018. Outcome model: *R*^2^ = 0.4467, *F*(3, 331) = 89.0624, *p* < 0.001. Total-effect model: *R*^2^ = 0.0289, *F*(2, 332) = 4.9352, *p* = 0.0077. Indirect-effect confidence intervals were estimated using 5,000 percentile bootstrap samples. Prior AI training was coded 0 = no and 1 = yes. ^*^Completely standardized indirect effect. Regression coefficients and standard errors are from the primary OLS model; the indirect-effect confidence interval is the percentile-bootstrap estimate from the same primary PROCESS model. HC3 results are reported separately as a robustness analysis. B = unstandardized coefficient; β = standardized coefficient; CI = confidence interval.

**Table 3:** Indirect association between AI literacy and positive attitudes toward AI through AI anxiety.

Model/path	*B*	SE/BootSE	*β*	*t*	*p*	95% CI/Bootstrap 95% CI
Mediator model: AI anxiety						
AI literacy → AI anxiety	−0.1775	0.0569	−0.169	−3.1205	0.0020	[−0.2894, −0.0656]
Prior AI training → AI anxiety	−0.1265	0.0912	−0.075	−1.3867	0.1665	[−0.3059, 0.0529]
Outcome model: Positive attitude						
AI literacy → Positive attitude	0.2674	0.0473	0.303	5.6499	<0.001	[0.1743, 0.3605]
AI anxiety → Positive attitude	0.0315	0.0450	0.037	0.6992	0.4849	[−0.0571, 0.1200]
Prior AI training → Positive attitude	−0.0717	0.0750	−0.051	−0.9559	0.3398	[−0.2193, 0.0759]
Total-effect model: Positive attitude						
AI literacy → Positive attitude	0.2618	0.0466	0.297	5.6168	<0.001	[0.1701, 0.3535]
Prior AI training → Positive attitude	−0.0757	0.0747	−0.053	−1.0127	0.3120	[−0.2227, 0.0713]
Indirect effect						
AI literacy → AI anxiety → Positive attitude	−0.0056	0.0112	−0.006^*^	–	–	[−0.0306, 0.0151]

*N* = 335. *B* = unstandardized coefficient; *β* = standardized coefficient; CI = confidence interval. Indirect-effect confidence intervals were estimated using 5,000 percentile bootstrap samples. Prior AI training was coded 0 = no and 1 = yes. ^*^Completely standardized indirect effect. Regression coefficients and standard errors are from the primary OLS model; the indirect-effect confidence interval is the percentile-bootstrap estimate from the same primary PROCESS model. HC3 results are reported separately as a robustness analysis.

As an additional sensitivity analysis, both models were re-estimated using an alternative AI literacy composite based on the nine non-reverse-worded items. This *post hoc* check assessed whether the main pattern depended on the three reverse-worded items and was not used to redefine the validated scale. To examine whether the findings depended on the specified cross-sectional ordering of the variables, further alternative-ordering sensitivity analyses were estimated using PROCESS Model 4 with 5,000 bootstrap resamples. These analyses evaluated plausible reversed sequences involving AI literacy, AI anxiety, and attitude scores. They were treated as sensitivity analyses rather than competing causal models because the cross-sectional design cannot establish temporal precedence.

## 3 Results

### 3.1 Descriptive statistics and relationships among variables

Table 4 presents descriptive statistics and Pearson correlations for the main variables. AI literacy was negatively correlated with AI anxiety (*r* = −0.178, *p* < 0.01), positively correlated with positive attitudes toward AI (*r* = 0.290, *p* < 0.01), and negatively correlated with negative attitudes toward AI (*r* = −0.150, *p* < 0.01). AI anxiety was strongly and positively correlated with negative attitudes toward AI (*r* = 0.667, *p* < 0.01). Despite this relatively strong association, the HTMT value between AI anxiety and negative attitudes was 0.746 (bootstrap 95% CI [0.667, 0.817]), supporting adequate discriminant validity between the two constructs. By contrast, AI anxiety was not significantly correlated with positive attitudes toward AI (*r* = −0.012, *p* > 0.05).

**Table 4:** Descriptive statistics and correlations among variables.

Variable	Mean	SD	Skewness	Kurtosis	1	2	3	4
1. AI literacy	5.20	0.73	−0.366	0.706	1			
2. AI anxiety	3.01	0.76	−0.138	−0.084	−0.178^**^	1		
3. Positive attitude toward AI	3.78	0.64	−0.831	2.211	0.290^**^	−0.012	1	
4. Negative attitude toward AI	3.23	0.81	−0.348	0.084	−0.150^**^	0.667^**^	0.111^*^	1

*N* = 335. ^*^p < 0.05; ^**^p < 0.01.

### 3.2 Indirect association between AI literacy and negative attitudes through AI anxiety

PROCESS Model 4 was used to estimate the indirect association between AI literacy and negative attitudes through AI anxiety. AI literacy was specified as the predictor, AI anxiety as the intervening variable, and negative attitude as the outcome, with prior AI training included as a covariate. The indirect effect was evaluated using 5,000 bootstrap resamples and a 95% bootstrap confidence interval.

As shown in Table 2, AI literacy was significantly and negatively associated with AI anxiety (*B* = −0.1775, SE = 0.0569, *t* = −3.1205, *p* = 0.0020), indicating that higher AI literacy was associated with lower AI anxiety. The AI-anxiety model was significant, *R*^2^ = 0.0372, *F*(2, 332) = 6.4179, *p* = 0.0018.

The negative-attitude outcome model was also significant, *R*^2^ = 0.4467, *F*(3, 331) = 89.0624, *p* < 0.001. AI anxiety was significantly and positively associated with negative attitudes toward AI (*B* = 0.6968, SE = 0.0441, *t* = 15.8088, *p* < 0.001). By contrast, the direct association between AI literacy and negative attitudes was not significant (*B* = −0.0328, SE = 0.0463, *t* = −0.7087, *p* = 0.4790). Prior AI training, included as a control variable, was also not significantly associated with negative attitudes (*B* = −0.0539, SE = 0.0735, *t* = −0.7335, *p* = 0.4638).

The total-effect model showed a significant negative association between AI literacy and negative attitudes toward AI (*B* = −0.1565, SE = 0.0604, *t* = −2.5903, *p* = 0.0100). After AI anxiety was included in the model, the direct association became non-significant, whereas the bootstrap indirect effect through AI anxiety was significant.

The estimated indirect effect through AI anxiety was *B* = −0.1237, and its 95% bootstrap confidence interval excluded zero (BootLLCI = −0.2065, BootULCI = −0.0398). This result indicates a significant statistical indirect association between AI literacy and negative attitudes through AI anxiety; it does not establish a causal sequence.

### 3.3 Indirect association between AI literacy and positive attitudes through AI anxiety

A second PROCESS Model 4 analysis estimated the indirect association between AI literacy and positive attitudes through AI anxiety. AI literacy was specified as the predictor, AI anxiety as the intervening variable, and positive attitude as the outcome, with prior AI training included as a covariate.

As shown in Table 3, the positive-attitude outcome model was significant, *R*^2^ = 0.0885, *F*(3, 331) = 10.7069, *p* < 0.001. AI literacy was significantly and positively associated with positive attitudes toward AI (*B* = 0.2674, SE = 0.0473, *t* = 5.6499, *p* < 0.001). By contrast, AI anxiety was not significantly associated with positive attitudes (*B* = 0.0315, SE = 0.0450, *t* = 0.6992, *p* = 0.4849). Prior AI training, included as a control variable, was also not significantly associated with positive attitudes (*B* = −0.0717, SE = 0.0750, *t* = −0.9559, *p* = 0.3398).

The total-effect model showed that AI literacy was significantly and positively associated with positive attitudes toward AI (*B* = 0.2618, SE = 0.0466, *t* = 5.6168, *p* < 0.001). The indirect effect through AI anxiety was not significant (*B* = −0.0056), and its 95% bootstrap confidence interval included zero (BootLLCI = −0.0306, BootULCI = 0.0151). Thus, there was no evidence of a statistical indirect association between AI literacy and positive attitudes through AI anxiety. A direct bootstrap contrast was then used to test whether the indirect effects for negative and positive attitudes differed significantly. The contrast between the negative-attitude indirect effect (*B* = −0.1237) and the positive-attitude indirect effect (*B* = −0.0056) was significant (contrast *B* = −0.1181, BootSE = 0.0415, bootstrap 95% CI [−0.2007, −0.0394]). Accordingly, the anxiety-linked indirect association was significantly more negative for negative attitudes than for positive attitudes.

### 3.4 Sensitivity analysis for reverse-worded AI literacy items

The *post hoc* sensitivity analyses were repeated using the mean of the nine non-reverse-worded AI literacy items. This exploratory composite showed good internal consistency (Cronbach’s alpha = 0.802; McDonald’s omega = 0.821). AI literacy remained negatively associated with AI anxiety (*B* = −0.1321, SE = 0.0522, *p* = 0.012). In the negative-attitude model, AI anxiety remained positively associated with negative attitudes (*B* = 0.7030, SE = 0.0439, *p* < 0.001), whereas the direct association between the nine-item AI literacy score and negative attitudes was not significant (*B* = 0.0068, SE = 0.0421, *p* = 0.872). The indirect association through AI anxiety remained significant (*B* = −0.0929, BootSE = 0.0425, bootstrap 95% CI [−0.1736, −0.0083]). However, the total association between the nine-item AI literacy score and negative attitudes was not significant (*B* = −0.0861, SE = 0.0555, *p* = 0.122).

In the positive-attitude model, AI literacy remained positively associated with positive attitudes (*B* = 0.2490, SE = 0.0429, *p* < 0.001), whereas AI anxiety remained unrelated to positive attitudes (*B* = 0.0242, SE = 0.0447, *p* = 0.588). The total association between AI literacy and positive attitudes remained significant (*B* = 0.2458, SE = 0.0424, *p* < 0.001), whereas the indirect association through AI anxiety remained non-significant (*B* = −0.0032, BootSE = 0.0087, bootstrap 95% CI [−0.0235, 0.0122]). Prior AI training was not significantly associated with the mediator or either attitude outcome in these sensitivity models. Thus, the principal indirect-effect pattern was retained, although the total association between AI literacy and negative attitudes was not robust to the alternative nine-item scoring.

### 3.5 Regression diagnostics and robustness checks

Regression diagnostics indicated no evidence of problematic multicollinearity, with VIF values ranging from 1.019 to 1.043 across the primary outcome models. Maximum Cook’s distances were well below 1, indicating that no single observation exerted excessive influence on the fitted models. Four observations in the negative-attitude model and three observations in the positive-attitude model had absolute studentized deleted residuals greater than 3.

The substantive findings were unchanged when HC3 heteroskedasticity-consistent standard errors were used. In the negative-attitude model, AI literacy remained negatively associated with AI anxiety (*B* = −0.1775, HC3 SE = 0.0607, *p* = 0.0037), AI anxiety remained positively associated with negative attitudes (*B* = 0.6968, HC3 SE = 0.0479, *p* < 0.001), and the bootstrap indirect effect remained significant (*B* = −0.1237, 95% CI [−0.2065, −0.0398]). In the positive-attitude model, AI literacy remained positively associated with positive attitudes (*B* = 0.2674, HC3 SE = 0.0507, *p* < 0.001), whereas AI anxiety remained unrelated to positive attitudes (*B* = 0.0315, HC3 SE = 0.0573, *p* = 0.5830); the indirect effect remained non-significant (*B* = −0.0056, 95% CI [−0.0306, 0.0151]).

The results were also robust to the temporary exclusion of observations with absolute studentized deleted residuals greater than 3. The indirect effect remained significant in the negative-attitude model (*B* = −0.1267, BootSE = 0.0447, 95% CI [−0.2114, −0.0377], *n* = 331) and non-significant in the positive-attitude model (*B* = −0.0017, BootSE = 0.0085, 95% CI [−0.0196, 0.0159], *n* = 332). Thus, the substantive conclusions did not depend on the identified residual outliers.

### 3.6 Alternative-ordering sensitivity analyses

Alternative-ordering analyses were conducted to evaluate whether the observed indirect associations were specific to the hypothesized cross-sectional ordering. When AI anxiety was specified as the predictor, AI literacy as the intervening variable, and negative attitude as the outcome, the indirect effect was not significant (*B* = 0.0053, BootSE = 0.0089, 95% CI [−0.0105, 0.0259]). In contrast, when AI literacy was specified as the predictor, negative attitude as the intervening variable, and AI anxiety as the outcome, the indirect effect was significant (*B* = −0.0966, BootSE = 0.0434, 95% CI [−0.1827, −0.0133]). A further reversed model, with positive attitude as the predictor, AI anxiety as the intervening variable, and AI literacy as the outcome, produced a non-significant indirect effect (*B* = 0.0025, BootSE = 0.0140, 95% CI [−0.0250, 0.0323]). These results indicate that the cross-sectional data do not uniquely identify the temporal ordering between AI anxiety and negative attitudes and reinforce the need to interpret the primary indirect-effect model as an associational decomposition rather than a causal sequence.

### 3.7 Evaluation of the hypotheses

Table 5 summarizes the support status of the research hypotheses based on the PROCESS analyses.

**Table 5:** Support status of the hypotheses.

Hypothesis	Result
H1: AI literacy is negatively associated with AI anxiety.	Supported
H2: AI anxiety is positively associated with negative attitudes toward AI.	Supported
H3: AI anxiety is negatively associated with positive attitudes toward AI.	Not supported
H4a: AI anxiety statistically accounts for a significant indirect association between AI literacy and negative attitudes toward AI.	Supported
H4b: AI anxiety statistically accounts for a significant indirect association between AI literacy and positive attitudes toward AI.	Not supported
H5: AI literacy is positively associated with positive attitudes toward AI.	Supported

The findings indicate that AI literacy was directly and positively associated with positive attitudes and showed a significant negative indirect association with negative attitudes through AI anxiety. No corresponding indirect association was found for positive attitudes. These results support H1, H2, H4a, and H5 but not H3 or H4b. They describe the specified cross-sectional statistical models and should not be interpreted as evidence of causal mediation.

## 4 Discussion

This study examined how AI literacy and AI anxiety were associated with positive and negative attitudes toward AI among preservice teachers. AI literacy was negatively associated with AI anxiety and positively associated with positive attitudes. AI anxiety was strongly and positively associated with negative attitudes but was not significantly associated with positive attitudes. Consequently, the bootstrap indirect association through AI anxiety was significant for negative attitudes but not for positive attitudes. The sensitivity analysis excluding the reverse-worded AI literacy items reproduced this pattern. Together, the findings are consistent with the possibility that positive and negative evaluations of AI have partly distinct cognitive and affective correlates. The strong association between AI anxiety and negative attitudes should nevertheless be interpreted cautiously because both constructs were assessed by self-report within the same questionnaire and both capture negatively valenced responses to AI. Although the HTMT analysis supported their empirical distinctness, shared method variance and some conceptual overlap in concern-related content may have contributed to the observed association.

The first main finding was that AI literacy was negatively associated with AI anxiety. This finding suggests that preservice teachers with greater knowledge, evaluative ability, and awareness of AI may experience less uncertainty about AI. Pei et al. (2025) identified gaps in preservice teachers’ understanding of AI mechanisms and highlighted the importance of practical experience, open educational resources, ethical awareness, and peer-based collaborative learning for developing AI literacy. From this perspective, the present finding suggests that AI literacy is not merely a cognitive competence; it may also be associated with affective responses toward AI.

Although the association between AI literacy and AI anxiety was statistically reliable, its standardized magnitude was modest (*β* = −0.169), and the mediator model explained only 3.7% of the variance in AI anxiety. By contrast, the association between AI anxiety and negative attitudes was much stronger (*β* = 0.659), and the negative-attitude outcome model explained 44.7% of the variance. AI literacy should therefore not be interpreted as a dominant explanation of AI anxiety.

This pattern has implications for teacher education. Limited knowledge of AI, difficulty understanding how AI systems work, and uncertainty about their educational consequences may be associated with higher anxiety, whereas greater AI literacy may coincide with more differentiated evaluations of these systems. This interpretation is consistent with Zhang et al. (2023), who identified perceived ease of use and perceived usefulness as key variables in preservice teachers’ acceptance of AI and showed that AI anxiety was related to some acceptance processes. Preservice teachers’ attitudes toward and acceptance of AI may therefore be related not only to access to technology but also to their understanding, evaluation, and perceived competence regarding that technology.

A second key finding was the positive association between AI anxiety and negative attitudes toward AI. AI anxiety may be related to concerns about job loss, loss of control, privacy, ethical uncertainty, algorithmic bias, and the weakening of the human role. Wang and Wang (2022) conceptualized AI anxiety as an anxiety response that may hinder individuals’ interaction with AI and identified dimensions such as learning, job replacement, sociotechnical blindness, and AI configuration. Hopcan et al. (2024) likewise reported that preservice teachers were particularly concerned about the effects of AI on employment and social life. In the present study, the positive association between AI anxiety and negative attitudes suggests that preservice teachers’ reservations about AI may have an affective basis.

The significant indirect association in the negative-attitude model is a central finding. Participants with higher AI literacy tended to report lower AI anxiety, and lower anxiety was, in turn, associated with lower levels of negative attitudes. Psychologically, greater understanding may coincide with reduced uncertainty and more differentiated risk appraisal. Nevertheless, the cross-sectional data cannot determine whether literacy precedes anxiety, whether anxiety shapes perceived literacy, or whether unmeasured factors influence both. The result therefore supports an anxiety-linked statistical pathway that should be tested longitudinally or experimentally rather than a confirmed causal mechanism. This caution is reinforced by the alternative-ordering analyses: although several reversed indirect effects were not supported, an alternative model in which negative attitude statistically intervened between AI literacy and AI anxiety also produced a significant indirect effect. Thus, the present data cannot determine whether anxiety precedes negative attitudes or negative attitudes precede anxiety.

These findings can also be considered alongside the technology acceptance literature. Davis (1989) identified perceived usefulness and perceived ease of use as key determinants of user acceptance, and Venkatesh and Davis (2000) argued that perceived usefulness is shaped by social influence and cognitive instrumental processes. The present study did not directly measure perceived usefulness or perceived ease of use; therefore, its findings cannot be mapped directly onto TAM variables. Nevertheless, the direct positive association between AI literacy and positive attitudes suggests that preservice teachers’ competence and understanding of AI may be related to perceiving the technology as more useful, understandable, or meaningful. This interpretation is broadly consistent with technology acceptance research linking knowledge, ease of use, usefulness, and attitudes.

Another key finding was the direct positive association between AI literacy and positive attitudes toward AI. Greater understanding and evaluative competence regarding AI were associated with more favorable appraisals of the technology. Al Darayseh (2023) showed that perceived benefits, ease of use, self-efficacy, attitudes, and behavioral intention were important in teachers’ acceptance of AI applications. Similarly, Zhang et al. (2023) identified perceived usefulness and perceived ease of use as key predictors of preservice teachers’ acceptance of AI applications. In the present study, the direct positive association between AI literacy and positive attitudes suggests that a clearer understanding of AI and its potential uses may accompany more favorable evaluations of the technology.

The non-significant association between AI anxiety and positive attitudes, together with the non-significant indirect effect for positive attitudes, requires careful interpretation. After accounting for AI literacy and prior training, anxiety was not statistically associated with participants’ positive evaluations. Preservice teachers may therefore hold concerns about AI while simultaneously maintaining positive evaluations of other aspects of the technology. This pattern supports treating positive and negative attitudes as distinct rather than as opposite endpoints of a single continuum. Schepman and Rodway (2020) distinguished benefit-related positive evaluations from concern-related negative evaluations, whereas Sindermann et al. (2021) differentiated acceptance and fear components. The present findings extend this distinction to preservice teachers, although replication is needed.

The theoretical contribution of the study lies in formally demonstrating an asymmetry between the two attitude dimensions. The direct bootstrap contrast showed that the anxiety-linked indirect association was significantly stronger for negative attitudes than for positive attitudes, while AI literacy retained a direct positive association with favorable evaluations. This pattern is compatible with a dual-evaluation account in which perceived benefits and affective concerns can coexist. Teacher-education content that demonstrates pedagogical affordances may therefore be more closely related to positive evaluations, whereas negative evaluations may additionally require explicit attention to uncertainty, job-replacement concerns, loss of control, privacy, ethical risk, and algorithmic transparency. These practical implications remain provisional until temporal and intervention-based evidence is available.

Prior AI training was included as a control variable but was not significantly associated with either positive or negative attitudes. This result suggests that prior AI training alone may not be sufficient to explain attitudes, but it should not be interpreted as evidence that AI training is ineffective. The variable used in this study did not capture the content, duration, quality, or practical nature of the training. Pei et al. (2025) emphasized the importance of practical experience, open and accessible resources, ethical awareness, and collaborative learning strategies for developing preservice teachers’ AI literacy. Lucas et al. (2025) likewise argued that preservice teachers were not fully ready for AI integration and that teacher education programs should be reviewed from this perspective. Accordingly, the non-significant role of prior AI training in the present study suggests that future research should examine the content and pedagogical quality of such training rather than only whether participants have received it.

## 5 Conclusion

AI literacy was associated with positive and negative attitudes toward AI in different ways. It showed a direct positive association with positive attitudes and a significant negative indirect association with negative attitudes through AI anxiety. The absence of a corresponding anxiety-linked indirect association for positive attitudes reinforces the value of treating positive and negative evaluations as related but distinct psychological dimensions.

These findings identify directions for future teacher-education research rather than demonstrating the effects of specific educational interventions. Longitudinal and experimental studies could examine whether learning experiences that develop AI literacy and demonstrate the pedagogical affordances and limitations of AI are associated with changes in positive evaluations. Similarly, intervention studies could test whether explicitly addressing uncertainty, job-replacement concerns, loss of control, privacy, ethical risks, and algorithmic transparency influences AI anxiety or negative attitudes toward AI. The present cross-sectional findings provide a rationale for testing these possibilities but do not establish that such interventions would produce the proposed changes.

The findings are consistent with viewing AI integration in teacher education as involving both competence-related and psychological dimensions. However, they do not show that increasing AI literacy will necessarily reduce anxiety or change attitudes. Longitudinal, experimental, and multi-institutional studies are needed to test temporal ordering, causal effects, and generalizability.

Because the measures assessed general AI-related constructs rather than pedagogical AI use specifically, the findings should not be interpreted as evidence about preservice teachers’ intentions to integrate AI into teaching or their evaluations of specific AI-supported instructional practices. Future research should directly assess pedagogical AI attitudes, integration intentions, and classroom-use behaviors to determine whether the present associations extend to instructional decision-making.

### 5.1 Limitations

This study has several limitations. First, its cross-sectional design does not establish temporal precedence; therefore, the reported indirect effects cannot support causal mediation claims or rule out alternative orderings of the variables. Second, all focal variables were measured through self-report in the same survey, creating the possibility of social-desirability, perceptual, and common-method bias. This concern is particularly relevant to the strong association between AI anxiety and negative attitudes because both constructs involve negatively valenced evaluations of AI. Although the HTMT result supported discriminant validity, it does not rule out shared method variance or partial overlap in item content. Third, the voluntary sample was drawn from a single faculty of education, which limits generalization to other institutions, programs, and cultural settings. Fourth, prior AI training was measured as a binary variable and did not capture its duration, quality, content, or practical emphasis. Fifth, the AI Literacy Scale showed modest internal consistency in this sample (*α* = 0.744; omega = 0.795), and item-level diagnostics suggested that two reverse-worded items may have contributed to the reduced internal consistency. Although the nine-item sensitivity analysis reproduced the main pattern, this *post hoc* check does not replace independent validation of the measurement structure. Future research should use longitudinal or experimental designs, multiple institutions, behavioral or performance-based indicators of AI literacy, and measurement models tested in independent samples.

## Data Availability

The de-identified participant-level analysis dataset, codebook, analysis syntax, statistical outputs, and data-cleaning documentation were provided confidentially with the revised submission for editorial and peer-review verification. The participant-level dataset is not deposited in an unrestricted public repository because the original informed-consent procedure did not include consent for public archiving or unrestricted secondary use of participant-level data. Additional information may be made available upon reasonable request, subject to applicable ethical requirements.
